# PKIDB: A Curated, Annotated and Updated Database of Protein Kinase Inhibitors in Clinical Trials

**DOI:** 10.3390/molecules23040908

**Published:** 2018-04-15

**Authors:** Fabrice Carles, Stéphane Bourg, Christophe Meyer, Pascal Bonnet

**Affiliations:** 1 Institut de Chimie Organique et Analytique (ICOA), UMR CNRS-Université d’Orléans 7311, Université d’Orléans BP 6759, 45067 Orléans CEDEX 2, France; fabrice.carles@univ-orleans.fr (F.C.); stephane.bourg@cnrs.fr (S.B.); 2 Janssen-Cilag, Centre de Recherche Pharma, CS10615-Chaussée du Vexin, 27106 Val-de-Reuil, France; cmeyer2@its.jnj.com

**Keywords:** protein kinase inhibitors, clinical trials, approved drugs, database, chemometrics analysis, kinome

## Abstract

The number of protein kinase inhibitors (PKIs) approved worldwide continues to grow steadily, with 39 drugs approved in the period between 2001 and January 2018. PKIs on the market have been the subject of many reviews, and structure-property relationships specific to this class of drugs have been inferred. However, the large number of PKIs under development is often overlooked. In this paper, we present PKIDB (Protein Kinase Inhibitor Database), a monthly-updated database gathering approved PKIs as well as PKIs currently in clinical trials. The database compiles currently 180 inhibitors ranging from phase 0 to 4 clinical trials along with annotations extracted from seven public resources. The distribution and property ranges of standard physicochemical properties are presented. They can be used as filters to better prioritize compound selection for future screening campaigns. Interestingly, more than one-third of the kinase inhibitors violate at least one Lipinski’s rule. A Principal Component Analysis (PCA) reveals that Type-II inhibitors are mapped to a distinct chemical space as compared to orally administrated drugs as well as to other types of kinase inhibitors. Using a Principal Moment of Inertia (PMI) analysis, we show that PKIs under development tend to explore new shape territories as compared to approved PKIs. In order to facilitate the analysis of the protein space, the kinome tree has been annotated with all protein kinases being targeted by PKIs. Finally, we analyzed the pipeline of the pharmaceutical companies having PKIs on the market or still under development. We hope that this work will assist researchers in the kinase field in identifying and designing the next generation of kinase inhibitors for still untargeted kinases. The PKIDB database is freely accessible from a website at http://www.icoa.fr/pkidb and can be easily browsed through a user-friendly spreadsheet-like interface.

## 1. Introduction

**Figure 1:**
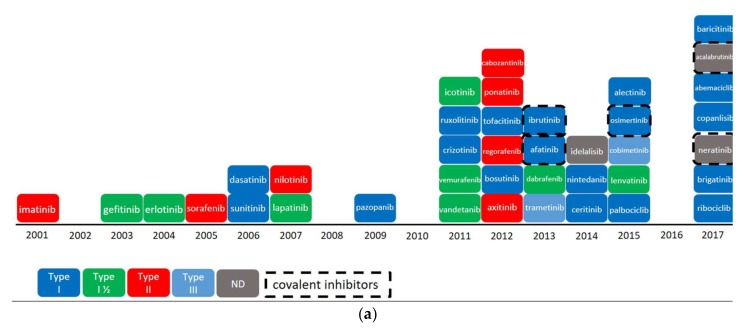

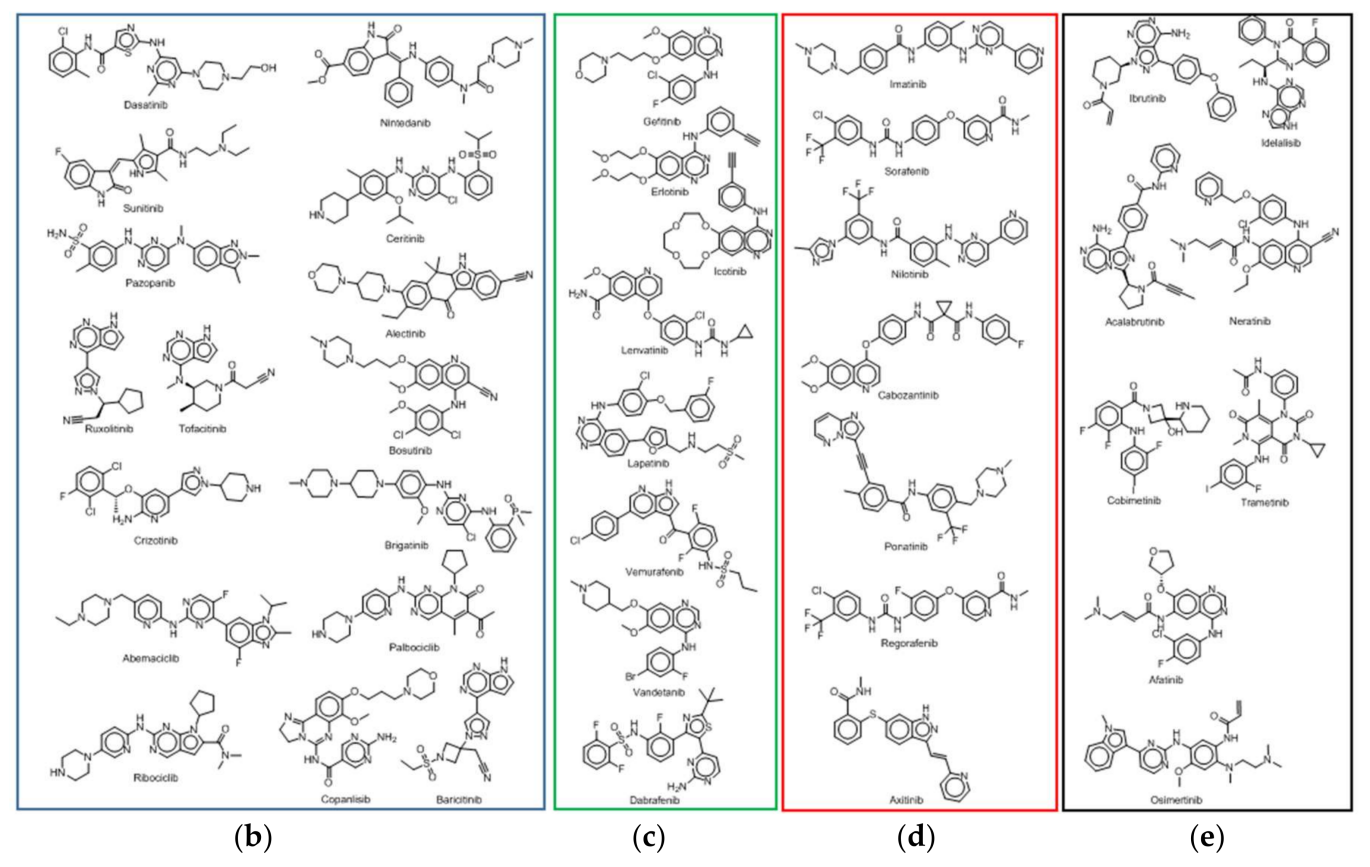
FDA-approved kinase inhibitors. Icotinib and baricitinib are approved by CFDA (China Food and Drug Administration) and by EMA (European Medicines Agency) respectively: (**a**) evolution of approved protein kinase inhibitors (2001–2017); (**b**) Structures of Type-I PKIs; (**c**) Structures of Type-I½ PKIs; (**d**) Structures of Type-II PKIs; (**e**) Structures of Type-I covalent inhibitors (ibrutinib, afatinib, osimertinib, neratinib, acalabrutinib), Type-III inhibitors (trametinib and cobimetinib) and PI3K lipid kinase inhibitor (idelalisib).

Protein kinases form one of the largest families of drug targets encoded by the human genome. Thirty-nine small protein kinase inhibitors (PKIs) were approved worldwide between 2001 and 2017. The Food and Drug Administration (FDA) has approved 37 of these, not counting macrocyclic lactones sirolimus, temsirolimus and everolimus (Figure 1a). Furthermore, icotinib and baricitinib received approval by the Chinese and European regulatory authorities for entering their respective markets. The vast majority of approved PKIs was released from 2001 when the first kinase inhibitor, imatinib, reached the market. This triggered a large number of research programs yielding a steady delivery of drugs. Surprisingly in 2016, for the first time in five years, no new kinase inhibitor was approved while seven PKIs were approved in 2017 worldwide. Several publications have already described the physicochemical properties and modes of binding of FDA-approved PKIs [1,2]. Likewise, a few databases recording data on PKIs are publicly available. As an example, the Kinase Profiling Inhibitor Database of University of Dundee [3] presents some bioactivity data for 243 commonly used signal transduction inhibitors. Robert Roskoski also compiled a small set of 39 FDA-approved protein kinase inhibitors [4]. However, these sources of information often focus on approved inhibitors only (compounds having reached phase 4 clinical trials) and do not provide long-term visibility on the next generation of PKIs. Extending the scope to PKIs currently in the clinic (compounds under development from phase 0 to phase 3) would bring useful information on the state of the art and current trends in kinase inhibitor design. Moreover, the databases mentioned above do not provide additional dynamic links to external databases or chemical information that could be exploited by chemoinformatics tools. Here, we have compiled the most comprehensive dataset on protein kinase inhibitors to date. We considered all protein kinase inhibitors whether approved or still in development phases and extracted relevant data available in the largest public chemical and clinical databases (see Materials and Methods section for details). By adding and updating monthly the dynamic links, our database represents a central hub to access any information related to PKIs. For example, the Protein Data Bank [5] ID of each PKI is collected and allows structural inspection, as well as annotation about the type of binding mode as soon as a new PDB structure is released. An analysis of the molecular determinants of PKIs as well as their chemical and biological target spaces could provide guidelines for the design of the next generation of PKIs. With this goal in mind, we collected the largest set of kinase inhibitors complying with the following criteria: compounds should be currently in one development phase (from Phase 0 to Phase 4), and each should have a disclosed chemical structure as well as an International Nonproprietary Name (INN). We found 180 PKIs fulfilling these criteria. Physicochemical and topological properties of these compounds were computed and related to their biological target profile. Data are stored in a database named PKIDB and are updated on a monthly basis. The database is freely accessible on a dedicated website (http://www.icoa.fr/pkidb) and can be browsed through a user-friendly spreadsheet-like interface. As of 1^st^ January 2018, it contained 180 inhibitors.

In order to classify the binding mode of kinase inhibitors (Figure 1b–e), we adopt a set of criteria described in the following section. For a detailed description of the binding mode, please refer to Roskoski’s review [6]. 

**Figure 2:**
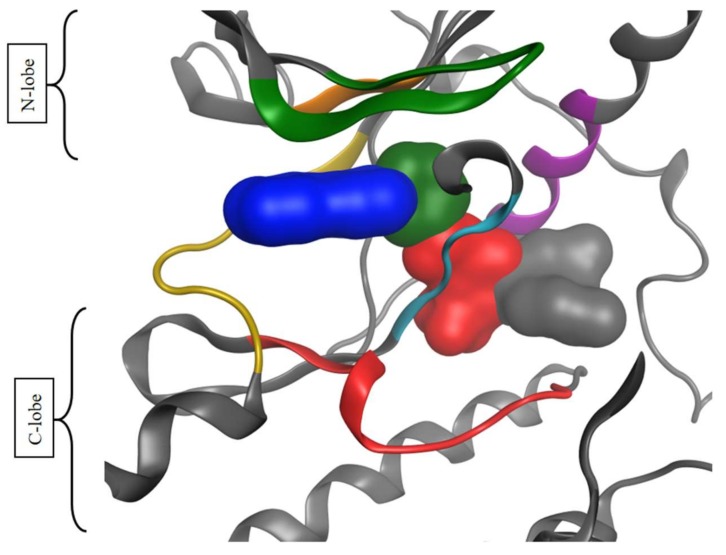
Representation of the different binding pockets targeted by kinase inhibitors. Blue, adenine binding site; green, hydrophobic pocket; red, allosteric pocket; and grey, adjacent allosteric pocket. The backbone of VEGFR2 (PDB ID 4ASD) is colored according to the following colors: yellow, hinge region; cyan, DFG motif; green, P-loop; purple, αC-helix; red, catalytic subunit; and orange, VAIK catalytic motif.

Protein kinases are enzymes that catalyze the transfer of the γ-phosphate group of ATP to the hydroxyl group of serine, threonine or tyrosine residues of protein substrates. The ATP binding site is located in the cleft separating the N-lobe from the C-lobe of the kinase domain. The adenine moiety of ATP (Figure 2, blue surface) binds a set of residues delineating the so-called ‘hinge region’ (Figure 2, yellow ribbon) and connecting the two lobes together. One hallmark of all kinases is the presence of an activation loop modulating the kinase activity. The activation loop starts with the three residues Asp-Phe-Gly (DFG motif) sequence and ends with the Ala-Pro-Glu (APE) motif. In the active conformation of the kinase, the phenylalanine residue of the DFG motif sits in a hydrophobic pocket in the ‘DFG-in’ conformation. When morphing from the catalytically active to the inactive conformation, the activation loop of a kinase undergoes a major conformational change putting the Phe in the ‘DFG-out’ conformation. This movement frees up an allosteric pocket (Figure 2, red and grey surfaces) adjacent to the ATP-binding site, also called the ‘back-pocket’. Kinase inhibitors are classified into categories according to their mode of binding to protein kinases. The first three types of inhibitors are ATP-competitive: Type-I inhibitors (Figure 1b) occupy the adenine binding site and bind the active form of the kinase (DFG-in). Type-II inhibitors (Figure 1d) depend on the ability of the protein to adopt a DFG-out conformation [7]. They usually extend in the adjacent allosteric pocket and stabilize the protein in an inactive conformation. Type-I½ inhibitors (Figure 1c) adopt an intermediate mode of binding between Type-I and Type-II. They bind the protein kinase in an active DFG-in conformation and extend towards the hydrophobic pocket (Figure 2, green surface) without occupying it as fully as Type-II inhibitors do [8]. Two additional types of inhibitors, Type-III and Type-IV (Figure 1e) are non-ATP competitive: Type-III inhibitors exclusively bind the allosteric pocket (back-pocket) adjacent to the ATP binding site in DFG-out (Figure 2, red and grey surfaces) or DFG-in (Figure 2, green surface) conformation of protein kinases. As observed in crystallographic structures, Type III MEK inhibitors bind the DFG-in conformation (Figure 2, extended green surface) in complex with ATP (PDB ID 4AN2) [9]. Finally, Type-IV inhibitors bind an allosteric pocket further away from the active site [10].

In addition, we can distinguish two categories of inhibitors: reversible and irreversible (or covalent-reversible) inhibitors. Reversible inhibitors are typical ATP-competitive PKIs binding protein kinases through non-bonded interactions. Irreversible inhibitors (Figure 1a,e) can covalently bind the receptor by exploiting a reactive nucleophilic cysteine proximal to the binding site. Using the above conventions, we manually annotated our PKI dataset using data from various public chemical databases. When available, crystallographic data were added, and visual inspection of the chemical structure was performed. This annotation allows us to compare the physicochemical properties of all protein kinase inhibitors already approved or currently under development and to identify some trends in the design of the next generation of kinase inhibitors.

## 2. Results

### 2.1. PKIDB Content and Interface

**Figure 3:**
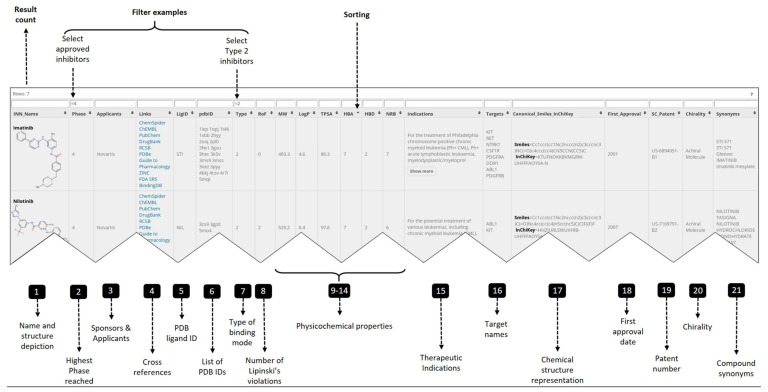
Overview of the PKIDB interface: Available data for each inhibitor are summarized over twenty-one columns. Filters above each column are provided to facilitate data visualization. Advanced searches can be performed by using operators and expression. Numerical <, ≤, >, ≥, =; String match =, *, !, {, }; Logical operator || (OR), && (AND). After filtering, the number of the remaining inhibitors is displayed in the top left corner. All fields of the table are sortable (alphabetically, numerically or both) by clicking on the column header (◆). In the first column, image upscaling is triggered by mouse hover.

The database (Figure 3) contains 180 PKIs described through twenty-one fields containing:

- Official INN name and synonyms for the PKIs (columns 1, 21)
- Compound representation: structure depiction, smiles and InChiKey (columns 1, 17)
- Clinical data: highest phase reached, therapeutic indication, first approval date (columns 2, 15, 18)
- Physicochemical properties (columns 8 to 14, 20)
- Applicants and patent id (columns 3, 19)
- Cross references (columns 4, 5)
- Target data: PDB ID, binding mode type, target name (columns 6 to 7, 16)

The PKIDB interface allows for browsing the data through a user-friendly spreadsheet-like interface. Columns can be filtered and sorted (Figure 3). Filters are composed of:

- Numerical filter: <, ≤, >, ≥ and =
- String match: exact match, =; partial match, *; match different search pattern, !
- Logical operators: OR, ||; AND, &&.

For convenience, string filters are not case sensitive and a selection like “novartis||bayer” or “Novartis||BAYER” in column Applicants (Figure 3, column 3) will match all inhibitors developed by the two pharmaceutical companies. A query example to select the seven approved Type 2 inhibitors is shown on Figure 3. More filtering examples are available online. In addition, sorting is available for all columns and can be used in combination with any filters. Ascending and descending sorting can be applied to sort alphabetically, numerically or alphanumerically. 

We have chosen to describe the compounds collected in our PKI dataset from different viewpoints, moving beyond the mere calculation of the classical physicochemical properties. In addition to computing the distribution and boundaries of these properties, we report hereafter the results of a Principal Component Analysis aimed at mapping the chemical space of the different inhibitor types. We also compared the topology (3D shape) of the PKIs under development to the topology of approved PKIs using a Principal Moments of Inertia analysis. Then, we mapped the PKIs under development on a kinome tree to allow the exploration at a glance of the protein kinase space targeted by these compounds. Finally, we tried to capture the trends and dynamism of the pharmaceutical sector by examining the clinical pipeline of the major players in the kinase field.

### 2.2. Physicochemical Analysis of the PKI Dataset

#### 2.2.1. Distribution of Physicochemical Properties of PKIs

A common quality check made on oral drug candidates consists of assessing their compliance with the Lipinski’s rule of five (Ro5) [11]. This rule states that a compound is more likely to show poor passive absorption or permeation if it violates two or more of the following constraints: molecular weight (MW) ≤ 500, calculated logP (ClogP) ≤ 5, number of hydrogen bond acceptors (HBA) ≤ 10, number of hydrogen bond donors (HBD) ≤ 5. Two additional properties, topological polar surface area (TPSA) and number of rotatable bonds (NRB), have also been shown to be correlated with oral bioavailability and the following conditions are often used in predictive drug design: TPSA ≤ 140 Å^2^ and NRB ≤ 10 [12]. These property ranges can serve as an early filtering strategy to reduce the size of a compound collection before running experimental or virtual screening campaigns.

Since current drugs do not always conform to Lipinski’s rules, it is expected that PKIs may also offend some of the four criteria proposed by Lipinski. We checked to which extent the compounds in the PKI dataset violate the Ro5 and found that a significant fraction of them have properties falling outside Lipinski’s boundaries (Table 1). Although 56% (or 63% depending on the method used to calculate the ClogP, Appendix A: Table A1) of the compounds fully comply with all conditions, almost one third of the compounds (28%) violate a single rule and 16% of the compounds violate two rules. These numbers are quite comparable to the statistics obtained for the approved PKI subset.

**Figure 4:**
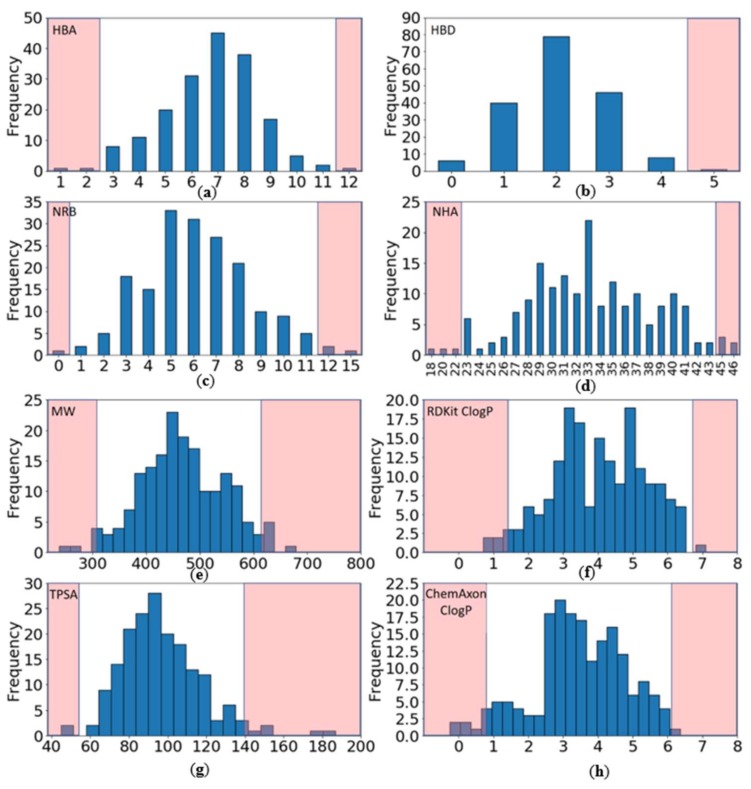
Distribution of physicochemical properties of PKIs: (**a**) Number of hydrogen bond acceptors (HBA); (**b**) Number of hydrogen bond donors (HBD); (**c**) Number of rotatable bonds (NRB); (**d**) Number of heavy atoms (NHA); (**e**) Molecular weight (MW); (**f**) ClogP (RDKit); (**g**) Topological polar surface area (TPSA); (**h**) ClogP (ChemAxon). Pink areas represent values outside two standard deviations from the mean (95.4% confidence interval).

Going into more details, we looked at the individual components composing Lipinski’s Ro5. Because the calculated logP is strongly dependent on the software used, the logarithm of the octanol/water partition coefficient was calculated using two different methods, the ClogP functionality included in the RDKit toolkit and the ClogP calculator from ChemAxon [13] (Figure 4f,h and Table 2 and Appendix A: Table A2). We found that most offending compounds exceed Lipinski‘s boundaries in terms of their molecular weight MW (32%) and their partition coefficient (ClogP, 24% or 12% using RDKit or ChemAxon respectively). In fact, high molecular weight and lipophilicity are often seen in Type-II inhibitors, whose chemical structure is elongated as compared to Type-I inhibitors. This is required when designing Type-II inhibitors, which extend to and interact within the kinase hydrophobic back-pocket but at the expense of a higher molecular weight. Interestingly, only one compound, barasertib, a prodrug of the active entity, contains five hydrogen-bond donor atoms and thus does not violate Lipinski’s rule.

As for TPSA and NRB properties (Table 3), the proportion of the PKIs complying with the criteria TPSA ≤ 140 Å^2^ and NRB ≤ 10 is very high. Only 3.9% and 4.4% of compounds exceed the TPSA and NRB thresholds, respectively. Similarly, all approved kinase inhibitors so far are compliant with these two criteria except lapatinib and neratinib.

The individual physicochemical properties follow a normal distribution as depicted by their bell-shaped curves (Figure 4). Only a few compounds have properties deviating significantly from the mean and can be seen as ‘exceptions to the rule’. In order to provide experimentalists with property ranges that apply to most kinase inhibitors, we disregarded property values beyond two standard deviations from the mean (95.4% confidence interval). The upper and lower molecular descriptor boundaries delimit the current chemical space of kinase inhibitors. They could be used as guidelines, rather than filters, to assist the prioritization of compounds with physicochemical properties comparable to current PKIs. It is important to note that these boundaries were extracted from protein kinase inhibitors being administrated orally. Thus, we could argue that the probability for a compound to reach clinical trials is greater if it does not violate most of these boundaries. The rational is that most of the existing approved and under-development kinase inhibitor drugs successfully passed the safety tests of Phase 1 by following theses boundaries (more than 90% of compounds in PKIDB have passed phases 0 and 1). 

Considering all PKIs, to be prioritized a compound could:

- Have a molecular weight (MW) between 309 and 617 Da (average of 463.3)
- Have a ClogP (calculated with RDKit) between 1.4 and 6.7 (average of 4.0)
- Contain between 0 and 4 hydrogen bond donors (HBD) (average of 2.1)
- Contain between 3 and 11 hydrogen bond acceptors (HBA) (average of 6.7)
- Have a topological polar surface area (TPSA) between 54 and 140 Å^2^ (average of 96.8)
- Contain between 1 and 11 rotatable bonds (NRB) (average of 6.2).

#### 2.2.2. Chemometrical Analysis of Protein Kinase Inhibitors

We asked ourselves whether PKIs have structural specificities that set this class of compounds apart from other orally bioavailable drugs. We reported previously that Type-II inhibitors tend to have a higher molecular weight and lipophilicity as compared to other types of kinase inhibitors [14]. This can likely be attributed to their binding mode requirement for an elongated structure, which is necessary to simultaneously bind hinge residues and fully occupy the adjacent hydrophobic back-pocket.

In order to gain insight into the relationships between the physicochemical properties of PKIs and their inhibitory effect on kinases, we mapped the PKI chemical space in a low dimensional space using Principal Component Analysis (PCA) [15]. PCA is a well-established multivariate statistical method able to condense a high-dimension description of individual entities, i.e., molecules in our case, into a 2D or 3D space. This space is delimited by factorial axes or principal components (PCs) formed by linear combinations of the original variables used to describe the individuals. The PCs are rank-ordered according to the fraction of the total variance accounted for by each. The graphs that are produced help understand similarities between molecules as well as correlations between variables (i.e., descriptors). It is routinely used in the chemoinformatics field to analyze chemical datasets [16].

We performed two separate PCAs on the following sets of compounds: the first set was built using the 180 PKIs collected in PKIDB augmented with 956 FDA-approved oral drugs. The goal was to determine whether the two classes of compounds could be efficiently discriminated into distinct groups; the second set contained only the 180 PKIs. Here, the goal was to highlight physicochemical features specific to each inhibitor type. Both compound sets were described using 11 classical physicochemical descriptors (Table 4) well suited to quantify chemical structures properties.

**Figure 5:**
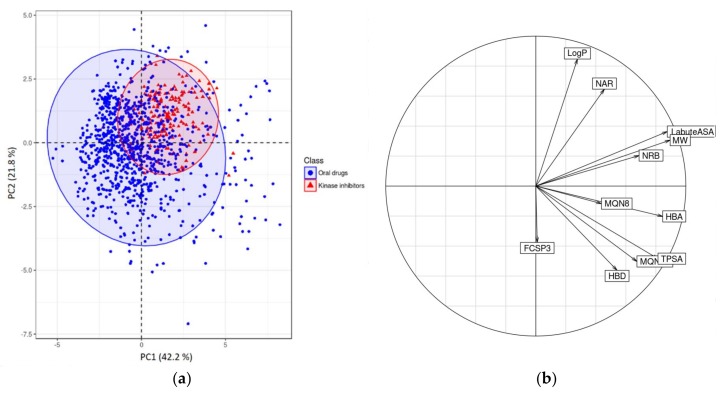
(**a**) PCA of FDA orally approved drugs and PKIDB containing 180 PKIs. Blue and red ellipses encompass 95% of the individuals from class “Oral drugs” and “Kinase inhibitors” respectively; (**b**) Corresponding correlation circle.

The first PCA plot (Figure 5) illustrates the chemical space of PKIs and oral drugs in a 2D space delimited by the two first principal components (PC1 and PC2).

The two first principal components explain 42.2% and 21.8%, respectively, of the total variance. This sums to 64%, which is an acceptable value for the graphical analysis of the data on a 2D scatterplot without losing too much information. The next PC (PC3) explains 13.8% of the total variance.

Each dot on the PC1/PC2 2D scatterplot represents a molecule. Molecules deviate from the center of gravity of the cloud (center of the graph if the initial data matrix was centered and reduced) by high values of the contributing descriptors of each factorial axis. PKIs occupy the top right quadrant of the graph while oral drugs tend to occupy the opposite top left and lower left quadrants. This suggests that PKIs share structural characteristics specific to this class of compounds. Sometimes a better separation can be achieved by accounting for the next component PC3, but this is not true here (data not shown).

Applying PCA on a correlation matrix enables the graphical representation of normalized variables in a unit hypersphere called ‘correlation sphere’ or ‘correlation circle’ in 2D representation (Figure 5b). In this space, collinear variable vectors are inter-correlated; likewise, vectors collinear to factorial axes are correlated with these axes. This allows assigning factorial axes a meaning in terms of original descriptors. A vector approaching the surface of the sphere (or circle in a 2D representation) indicates a strong contribution to the creation of a factorial axis.

Analysis of the correlation circle (Figure 5b) shows that the first factorial axis is correlated with high MW and Labute’s Approximate Surface Area (LabuteASA). These two variables contribute to PC1 with values of 17.2% and 16.5%, respectively. The second axis, PC2, is correlated with a high number of aromatic rings (NAR) and high logP (contribution of 17.4% and 29.9% respectively), and negatively correlated with the fraction of sp3 hybridized carbon atoms (FCSP3). These observations support the fact that kinase inhibitors are known to be less flexible than other drugs and contain more aromatic rings. Collinearity between NAR and logP is consistent with the fact that logP increases mechanically with the number of aromatic rings. The correlation with the molecular weight confirms the preliminary observation inferred from the distribution of the physicochemical properties of the PKIs; they are bigger molecules than the average of oral drugs and have higher LabuteASA.

**Figure 6:**
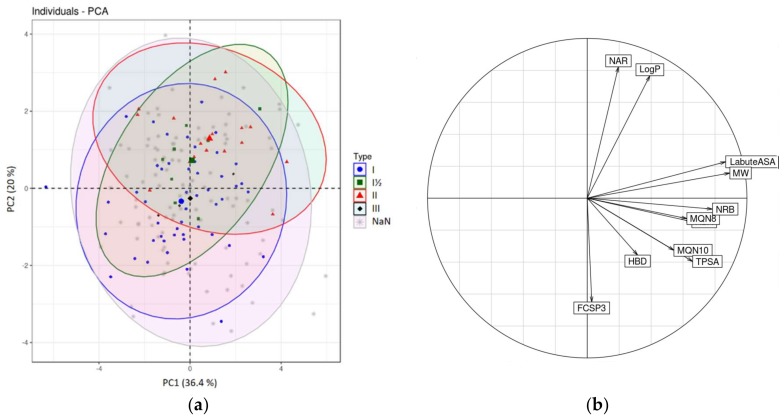
(**a**) PCA of PKIs colored by inhibitor type. Ellipses encompass 95% of individuals of each respective inhibitor type except for Type-III inhibitors because there were too few data points. “NaN” is also defined as “unknown kinase inhibitor Type”; (**b**) Corresponding correlation circle.

The second PCA plot (Figure 6) is a projection of the PKI dataset in the PC1/PC2 factorial plane. PKIs are labelled according to their type (Type-I, Type-I½, Type-II, Type-III and NaN for unknown kinase inhibitor Type).

The variance explained by the three first principal components PC1, PC2 and PC3 is 36.4%, 20% and 14%, respectively (56.4% explained by PC1 and PC2 alone). The most contributing variables to PC1 are, in decreasing order of importance, the molecular weight (19.9%), Labute’s approximate surface area (18.8%) and the number of rotatable bonds (15.4%). Thus, PC1 primarily represents molecular size. For PC2, the most contributing variables are the number of aromatic rings (30.8%), logP (26.8%), and the fraction of sp3 hybridized C atoms (19.2%).

In this plot, inhibitors were colored according to their type of binding mode when the information was available. As expected, PC1 is not able to discriminate inhibitor types, but rather large and flexible molecules from smaller compounds. Type-I and Type-II inhibitors, however, are better separated along the PC2 axis as is apparent from their projection in two different areas of the plot. Indeed, PC2 is correlated with the same variables as those able to discriminate PKIs from oral drugs in our first PCA analysis (Figure 5). Type-II inhibitors contain more aromatic rings and are more planar than Type-I inhibitors. They need to reach the adjacent hydrophobic pocket, which tolerates aromatic moieties. On the other hand, Type-I inhibitors generally contain a greater fraction of sp3 carbons, which is consistent with the negative correlation of FCSP3 with PC2. Type-I½ inhibitors are distributed evenly across Type-I and Type-II chemical spaces. Because of their hybrid nature, they combine Type-I and Type-II features and display physicochemical properties similar to both types of inhibitors. The three Type-III inhibitors (trametinib, cobimetinib and selumetinib) that bind specifically to the allosteric pocket are projected in the center of the PCA plot and cannot be discriminated by the first two principal components.

#### 2.2.3. Principal Moments of Inertia

**Figure 7:**
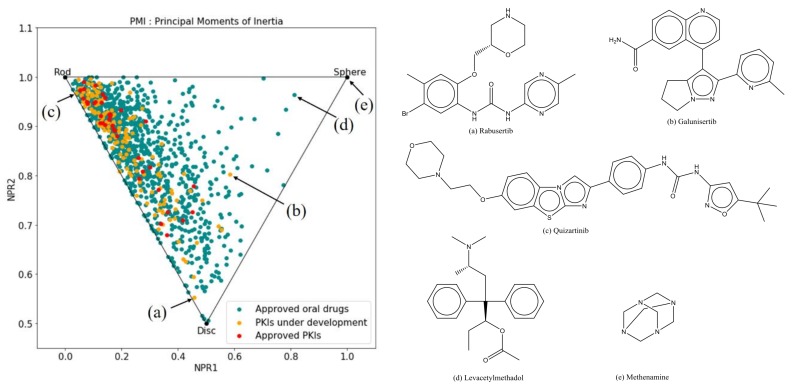
Principal Moments of Inertia (PMI) plot of orally approved drugs (in green) and PKIs (in yellow and red). Compounds **a**, **b** and **c** represent the extreme shape of the PKI dataset. Compounds **d** and **e** are the most spherical oral drugs.

The Principal Moments of Inertia (PMI) plot visually represents the shape-based distribution of a set of molecules [17]. In a PMI plot, molecules are projected in a triangular space (Figure 7) with its vertices representing the extremes of molecular shape: rod (diacetylene), disc (benzene) and sphere (adamantane). Here, we used this method to simultaneously compare the shape diversity of three sets of compounds: approved PKIs, PKIs under development and other oral drugs. Since the shape of a ligand is often complementary to the shape of a protein binding site, molecules spanning a wide space of the PMI plot are expected to target a large diversity of protein sites. Conversely, ligands occupying a narrow shape space of the PMI plot could target similar binding sites like the ATP binding site of kinases, for instance.

As apparent in Figure 7, the space covered by the structurally-diverse oral drugs (green-blue) is wider than the one occupied by both PKI sets (red and orange). The distribution of oral drugs is skewed to elongated and circular shapes. A few drugs, however, exhibit a spherical-like shape. The most spherical drug is methenamine (**e**), used as an antibacterial drug for the treatment of urinary tract infection. This drug shows up in the same location as adamantane because of its similar “cage-like” 3D structure. Levacetylmethadol (**d**) is also an example of a drug that adopts a spherical shape because of its central stereogenic center that is able to project substituents in all directions in space.

Most kinase inhibitors are located close to the rod vertex and along the popular rod-disc axis. Type-II PKIs such as quizartinib (Figure 7c) can be found close to the rod edge because of their binding requirement for an extended conformation [18,19]. The three PKIs closest to the extreme vertices are all molecules under development such as rabusertib (**a**), galunisertib (**b**), and quizartinib (**c**) (Figure 7). This might be an indication that kinase inhibitors currently in development tend to exhibit new molecular shapes and potentially novel chemical space by moving away from the rod-disc axis.

### 2.3. Overview of Current Targeted Protein Kinases

Annotation of the kinome phylogenetic tree has become a popular way of analyzing the protein kinase space at a glance. Here, we were interested in mapping the kinases targeted by PKIs (approved or still in development) across all kinome groups. Targeted kinases are represented by color-coded dots of varying size. Color and size relate to the highest phase reached by PKIs and to the number of PKIs targeting a particular kinase, respectively.

**Figure 8:**
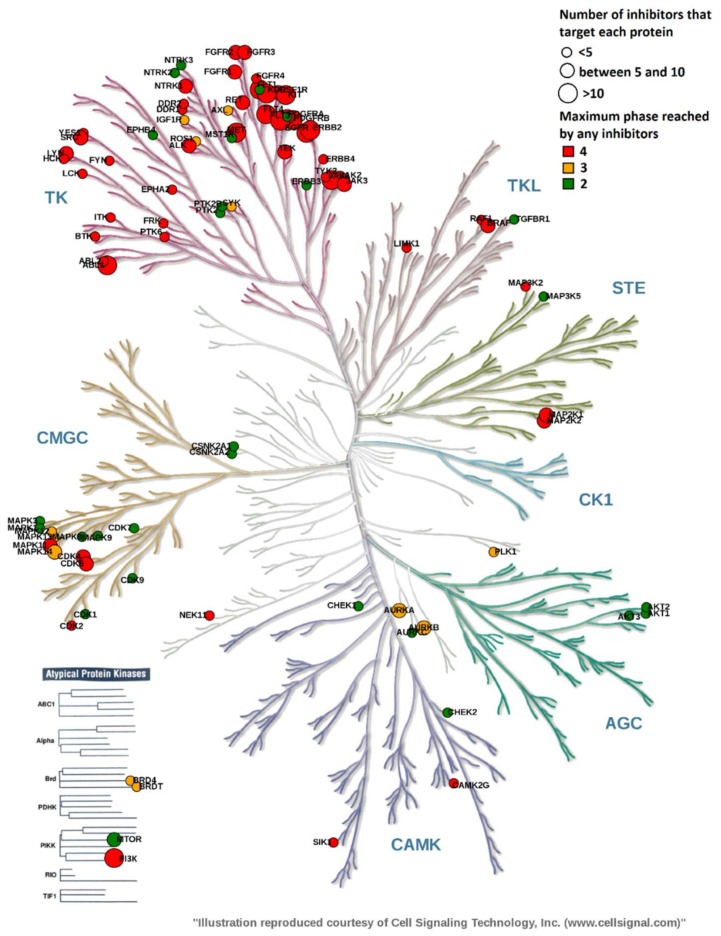
Kinome tree exploring the protein kinase space. The maximum phase reached by an inhibitor is color-coded: phase 4 (red), phase 3 (orange) and phase 2 (green). The size encodes the number of PKIs per protein kinase: small size encodes a number of inhibitors less than 5, medium size encodes a number of inhibitors between 5 and 10 and large size encodes a number of inhibitors greater than 10.

As expected, the annotated kinome tree shows that the tyrosine kinase (TK) group is by far the most targeted group (Figure 8). ABL, EGFR, KIT, PDGFR and FLT3 kinases have been the primary targets for the development of PKIs with 10 or more inhibitors having reached the market.

The other kinase groups are less represented by PKIs: The TKL, STE, CAMK and CMGC groups count less than five approved inhibitors per group while groups CK1 and AGC did not yield any marketed drug yet. Ribociclib, vemurafenib, cobimetinib and bosutinib target CMGC, TKL, STE and CAMK groups respectively. On the other hand, several candidates, like uprosertib, have reached Phase 2 clinical trials, specifically targeting the AKT sub-family. For all groups, except CK1, small molecule inhibitors are actively pursued in development, some currently in Phase 3.

Regarding the atypical protein kinase family, most inhibitors target the PIKK family; idelalisib is the first approved inhibitor against the PI3Kδ isoform. The three drugs sirolimus, temsirolimus and evelorimus approved respectively in 1999, 2007 and 2009, were not considered in this study since they do not target directly the mTOR kinase but rather an upstream protein. For instance, sirolimus (also called rapamycin) works through binding the immunophilin FK binding protein 12 (FKBP12) to generate an immunosuppressive complex able to inhibit the activation of mTOR.

### 2.4. Worldwide Pharmaceutical Companies Pipeline Overview of Kinase Inhibitors

**Figure 9:**
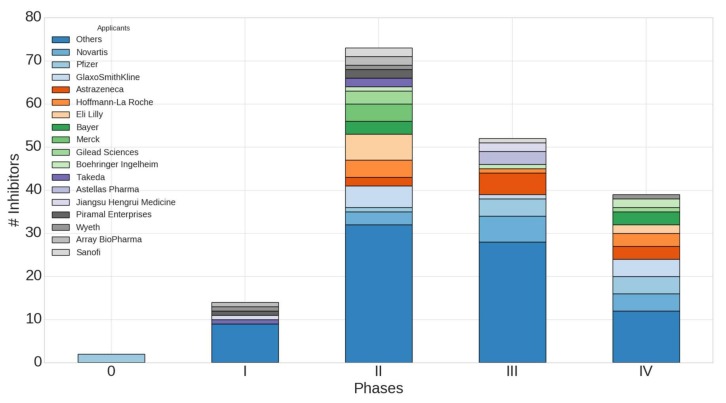
Protein kinase inhibitors having reached the highest phase of clinical trials grouped by pharmaceutical companies.

PKI clinical data could be extracted from the clinicaltrials.gov database. We queried the database using INN or generic name information of the PKIs in development or already on the market. Since most of the drugs receive an INN when they reach late-phase clinical studies, the group of PKIs in Phase 1 is most probably underrepresented. Figure 9 represents the distribution of the PKIs that we have collected, grouped by development phase and pharmaceutical company. It is important to note that the phases mentioned in Figure 9 represent the highest phase reached by a drug on any market. For instance, 37 protein kinase inhibitor drugs reached the U.S. market (FDA-approved drugs) but two other drugs, icotinib and baricitinib, entered only the Chinese and European market, respectively. For more clarity, pharmaceutical companies with less than three PKIs in their pipeline have been merged in the category “others”. Thus, this category contains the largest number of kinase inhibitors.

Seventy-three drugs are currently evaluated in Phase 2, with Eli Lilly, GlaxoSmithKline, Hoffmann-La-Roche, Novartis, Bayer Gilead Sciences and Merck being strongly represented. Fifty-two kinase inhibitors have been found in Phase 3 clinical trials with Novartis being the company with the largest number of PKIs under investigation followed by Pfizer, AstraZeneca, GlaxoSmithKline and Eli Lilly in the top five. As for Phase 4, the first three pharmaceutical companies are GlaxoSmithKline, Novartis and Pfizer. These companies have delivered promising kinase inhibitors that will, hopefully, provide novel medical solutions to patients. 

We can conclude that with 52 kinase inhibitors in Phase 3 clinical trials, the future of protein kinase inhibitors is still promising. While no PKI was approved in 2016, 7 kinase inhibitors were approved in 2017.

## 3. Discussion

PKIDB compiles all marketed kinase inhibitors to date as well as kinase inhibitors currently in clinical trials. Only compounds that received an INN have been included in the database. Because INNs are usually requested after clinical development has been initiated, it is very likely that some compounds currently in development phase I are missing from our database. Presently, PKIDB contains 180 compounds along with annotations from multiple chemical–protein resources (see Material and Methods for details).

The PKI dataset was analyzed from different perspectives to highlight features specific to these compounds. Calculation of physicochemical properties demonstrates that more than one third of the compounds violate at least one Lipinski’s rule. The Rule of Five was derived from examination of the property distribution of existing orally-administrated drugs on the market before 2000s. While imatinib, the first PKI approved in 2001, complies with all rules, several kinase inhibitors that reached the market later have some molecular properties exceeding these limits. From this analysis, we propose new boundaries (minimum, maximum, average) reflecting the properties of the global PKI population at a 95% confidence level. This convention coincides with the 5% convention of statistical significance in hypothesis testing. It allows for not overestimating the influence of outliers exhibiting extreme properties. A good practice would be to use these conditions as flags to prioritize compounds rather than as hard-cutoffs to downsize a screening compound collection. Because we compute these properties on orally approved and under development PKIs it is possible that they are not suited for other applications such as parenteral route of administration. For example, antibiotic or antifungals PKIs target non-human protein kinases and their mode of administration could be different.

The comparison with oral drugs also advocates for distinctive features of PKIs. The mapping of PKIs in the chemical space resulting from PCA transformation shows that PKIs occupy a distinct region in that space driven by high values of molecular weight, lipophilicity and aromatic content. Indeed, PKIs are generally larger molecules with higher logP and predominantly aromatic framework as compared to more “classical” oral drugs. This is particularly significant for Type-II inhibitors, whose structure must comply with shape, chemical content and conformation requirements needed to lock the kinase protein in the DFG-out inactive conformation. Pharmaceutical companies screening decks still contain many achiral, aromatic compounds as a result of past high-throughput synthetic practices. Gradual introduction of carbon bond saturation and stereogenic centers may help escaping from “flat land” and securing new intellectual property (IP) of future PKIs [20].

The PMI analysis reveals that PKIs cover a narrower shape diversity as compared to oral drugs. One obvious explanation of this behavior comes from the nature of the two sets of compounds being compared: while oral drugs have shapes complementing a variety of binding pockets topologies, PKIs have been designed to target the highly conserved kinase domain. Although most kinases show an intrinsic plasticity, their binding site topology is remarkably conserved whether the protein is in DFG-in or in DFG-out conformation. Regarding the PKI dataset alone, approved PKIs cluster tightly around the rod edge with few exceptions whereas PKIs under development tend to explore wider territories. More frequent macrocyclization has certainly contributed to the drift toward the disc edge. Another reason for that trend is that next-generation kinase inhibitors need to escape a crowded IP space while being as selective as possible. Therefore, the design of new chemically diverse structures is highly desirable.

The annotated kinome tree provides a visual overview of the kinase space targeted by approved PKIs and PKIs currently in development. Because we focused on PKIs having received an INN name, inhibitors identified by their research code in the clinicaltrials.gov database were not considered and, therefore, do not appear on the kinome tree. The most targeted family is the Tyrosine Kinase group (TK) while the Casein Kinase 1 group (CK1) stays untouched. TKs have been shown to play a critical role in cancer biological pathways [21]. The successful discovery and development of imatinib proved that TKs are druggable proteins and has triggered many discovery programs, some leading to approved PKIs. The annotated kinome tree shows that PKIs are being developed for kinases pertaining to most kinase groups.

Examination of the pharmaceutical industry pipeline shows that 73 and 52 kinase inhibitors are currently evaluated in Phase 2 and Phase 3 clinical trials, respectively. All major companies have active research programs in the kinase field, and strategies have been put in place to reduce the attrition rate as much as possible [22]. For instance, using predictive biomarkers to select patients with known molecular aberrations for specific kinase inhibitors has been shown to reduce attrition. Seven kinase inhibitors were approved in 2017, and the future of kinase research looks promising for the benefit of patients.

The PKIDB database can be accessed through a website page available at http://www.icoa.fr/pkidb. It can be browsed using a spreadsheet-like interface and is monthly updated. We hope that this resource will assist researchers in their quest for novel kinase inhibitors.

NB: since the analysis of PKIDB (version December 2017), 2 new kinase inhibitors (capivasertib, and neflamapimod in phase 2 clinical trials) were added to PKIDB, resulting in the database consisting of a total of 182 PKIs.

## 4. Materials and Methods

Since 1953, when the first INN list for pharmaceutical substances was published by the World Health Assembly [23], a cumulative list of around 8800 names provides a unique and universally available designated name to identify each pharmaceutical substance. An important feature of the INN system is that the names of pharmacologically-related substances demonstrate their relationship by using a common “stem”. Each stem helps the community in medical and pharmaceutical areas to recognize that the substance belongs to a group of substances having similar pharmacological activity. Here, we have collected the entire set of protein kinase inhibitors with known INN by filtering data retrieved from several sources including the ChEMBL DrugStore [24] and clinicaltrials.gov [25]. Missing structures and additional information were retrieved later using ChemSpyder [26], Unichem [27], DrugBank [28], PDB [5] and Guide to Pharmacology [29]. Then, a manual annotation and curation of structure and data were performed. First, using a blacklist we removed some erroneous INN like “afatanib” or “idelisib” which should be spelled afatinib and idelalisib, respectively. Then, we manually checked chemical structures, FDA approval of new PKIs and added indications. The name of pharmaceutical companies that develop each drug candidate was manually collected. We manually annotated the inhibitor type based on previous work from the Möbitz dataset [30] and completed by visual inspection of co-crystal structure when available. The targeted kinase of each molecule was manually collected when no target was provided in DrugBank. Then, the kinome tree was produced from the KinMap webservice [31]. Since the annotations of atypical kinases is not yet fully implemented in the KinMap webservice, some kinases like PI3K were manually added to the kinome tree. Indeed, PI3K is not classified in Manning’s kinome tree [32] but is well identified as a member of PIKK family in the UniProt—Swiss-Prot Protein Knowledgebase pkinfam file http://www.uniprot.org/docs/pkinfam.

To be digitally exploited, several phase annotations of clinical studies were converted in terms of numbers: For example, “Phase1/Phase2” clinical studies were converted to phase “1.5”. The descriptor calculations were performed with RDKit version 2017.09.2, except for the Principal Moment of Inertia (PMI) for which we used MOE version 2016.08 (Molecular Operating Environment, Chemical Computing Group, Inc., Montreal, QC, Canada) [33]. Three compounds (diacetylene, benzene and adamantane) were added to the PKIs dataset as reference shapes to delimit the PMI triangle. For all molecules, an energy minimization was performed in MOE before PMI calculation using the MMFF94x forcefield. Calculator Plugins Marvin 15.2.9.0, 2015, ChemAxon (http://www.chemaxon.com) [13] were used for additional logP calculations as well molconvert for 2D molecule depiction in the website http://www.icoa.fr/pkidb. Two PCAs were performed in R with the ade4 [34] and factoextra packages version 1.7.6 and 1.0.4, respectively. We used a set of 11 physicochemical descriptors (Table 4) known to be well suited to describe the chemical diversity in PCA reduction analysis. 

In the first PCA, we collected 974 approved oral drugs by removing PKIs, salts, and molecules with a phase <4 from the ChEMBL drugstore [12]. A manual suppression of outliers was done after PCA projection of the 974 molecules in PC1 and PC2. Eighteen outliers were removed, and the final projection contained 956 oral approved drugs and 180 kinase inhibitors. An outlier is a molecule characterized by an extreme value in some variables and therefore results in an excessive contribution to the PCA that biases the projection. The second PCA was conducted after manual annotation of inhibitor type in order to highlight the chemical diversity in inhibitor type.

Except for PCA, all figures are made using Python Matplotlib package version 2.1.1. Figure 2 uses the backbone of the VEGFR2 receptor (PDB ID 4ASD) as a template and was created in MOE.

## Figures and Tables

**Table 1:** Comparison of Lipinski’s rules violation between approved and in clinical trials PKIs.

^1^	No Ro5 Violation	1 Ro5 Violation	2 Ro5 Violations
PKIs approved	20/39 (51%)	11/39 (28%)	8/39 (21%)
PKIs in clinical trials	80/141 (57%)	40/141 (28%)	21/141 (15%)
All PKIs	100/180 (56%)	51/180 (28%)	29/180 (16%)

^1^ RDKit was used to calculate all descriptors including ClogP.

**Table 2:** Number of PKIs violating at least one Lipinski rule.

	MW > 500 Da	ClogP > 5 ^1^	HBA > 10	HBD > 5
PKIs approved	13/39 (33%)	11/39 (28%)	1/39 (2.5%)	0/39 (0%)
PKIs in clinical trials	44/141 (31%)	32/141 (23%)	2/141 (1.4%)	0/141 (0%)
All PKIs	57/180 (32%)	43/180 (24%)	3/180 (1.7%)	0/180 (0%)

^1^ RDKit was used to calculate all descriptors including ClogP.

**Table 3:** Number of PKIs violating at least one Veber rule [12].

^1^	TPSA > 140 Å^2^	NRB > 10
PKIs approved	0/39 (0%)	2/39 (5.1%)
PKIs in clinical trials	7/141 (5%)	6/141 (4.3%)
All PKIs	7/180 (3.9%)	8/180 (4.4%)

^1^ RDKit was used to calculate all descriptors.

**Table 4:** Descriptors used for PCA.

Name Variable	Descriptor
MW	Molecular weight
LogP	Wildman-Crippen LogP value
TPSA	Topological polar surface area
HBA	Number of Hydrogen Bond Acceptors
HBD	Number of Hydrogen Bond Donors
NRB	Number of Rotatable Bonds
LabuteASA	Labute’s Approximate Surface Area
NAR	Number of aromatic rings
FCSP3	Fraction of C atoms that are sp3 hybridized
MQN8	Molecular QuantumNumbers
MQN10	Molecular QuantumNumbers

## Appendix A

**Table A1:** Comparison of violation of Lipinski’s rules between approved and in0clinical trial PKIs.

^1^	No Ro5 Violation	1 Ro5 Violation	2 Ro5 Violations
PKIs approved	25/39 (64%)	10/39 (26%)	4/39 (10%)
PKIs in clinical trials	89/141 (63%)	41/141 (29%)	11/141 (7.8%)
All PKIs	114/180 (63%)	51/180 (28%)	15/180 (8%)

^1^ ChemAxon was used to calculate ClogP and RDKit for the remaining descriptors.

**Table A2:** Number of PKIs violating at least one Lipinski rule.

	MW > 500 Da	ClogP > 5 ^1^	HBA > 10	HBD > 5
PKIs approved	13/39 (33%)	4/39 (10%)	1/39 (2.5%)	0/39 (0%)
PKIs in clinical trials	44/141 (31%)	17/141 (12%)	2/141 (1.4%)	0/141 (0%)
All PKIs	57/180 (32%)	21/180 (12%)	3/180 (1.7%)	0/180 (0%)

^1^ ChemAxon was used to calculate ClogP and RDKit for the remaining descriptors.
